# Supplementary material for: Deep Learning-Based Classification of Uterine Cervical and Endometrial Cancer Subtypes from Whole-Slide Histopathology Images
Source: Diagnostics (Basel). 2022 Oct 28;12(11):2623. doi: 10.3390/diagnostics12112623 (PMC9689570; doi:10.3390/diagnostics12112623)
Supplement: Supplementary file 1 [file diagnostics-12-02623-s001.zip › Supplementary Table S1.pdf]

Supplementary Table S1-1. Numbers of tissue image patches for the normal/tumor tissue classifiers. Training epoch was also presented.

	Number of normal image patches	Number of tumor image patches	Training epoch
TCGA-CESC	539,817	683,291	16
TCGA-UCEC	613,784	736,988	13

Supplementary Table S1-2. Average numbers of tissue image patches in each training fold for the five-fold cross validation scheme to train the classifiers for the subtypes and origin of cancers. The average training epoch was also presented.

Cervical cancer subtypes classification	Average patch number for cervical squamous cell carcinoma	Average patch number for endocervical adenocarcinoma	Average training epoch
	117,438	78,465	18
Endometrial cancer subtypes classification	Average patch number for endometrioid endometrial adenocarcinoma	Average patch number for serous endometrial adenocarcinoma	Average training epoch
	387,155	264,531	13
Tumor origin classification	Average patch number for endocervical adenocarcinoma	Average patch number for endometrioid endometrial adenocarcinoma	Average training epoch
	78,465	118,197	14
