# Supplementary material for: Deep Learning-Based Classification of Uterine Cervical and Endometrial Cancer Subtypes from Whole-Slide Histopathology Images
Source: Diagnostics (Basel). 2022 Oct 28;12(11):2623. doi: 10.3390/diagnostics12112623 (PMC9689570; doi:10.3390/diagnostics12112623)

Supplementary Figure S1. Schematic view of the network structure for the classification of tissue subtypes. First, whole slide images were tessellated into 360×360 pixel image patches. Then, the image patches were provided as inputs to the Inception v3 network for classification. The last layer of the Inception v3 network is a softmax layer for 2-class classification in the present study.

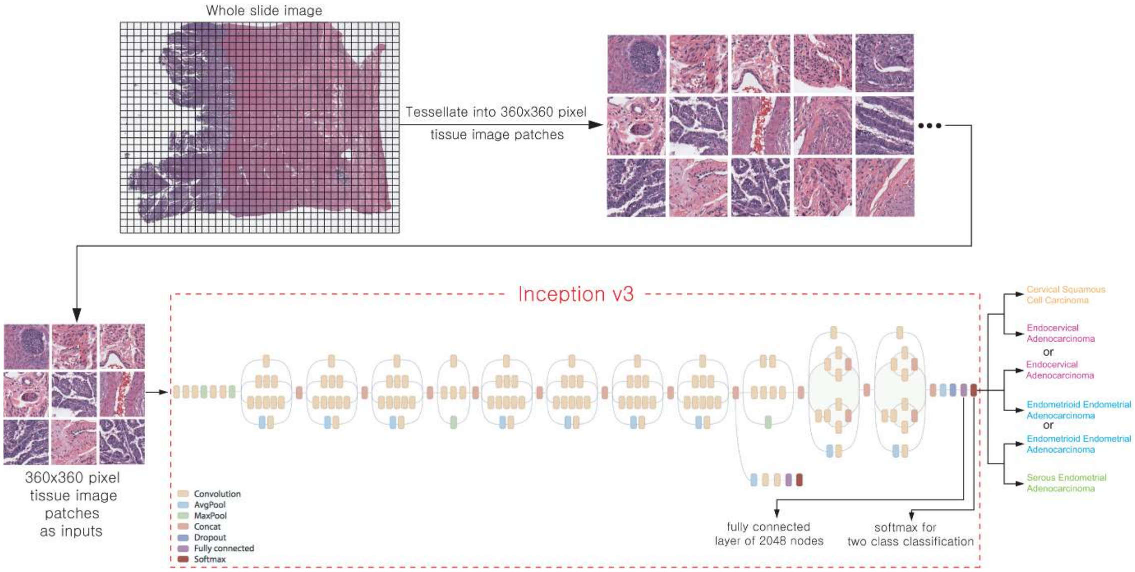

Supplement: Supplementary file 1 [file diagnostics-12-02623-s001.zip › Supplementary Figure S1.pdf]
